# Treatment of the benign bone tumors including femoral neck lesion using compression hip screw and synthetic bone graft

**DOI:** 10.1051/sicotj/2015009

**Published:** 2015-06-26

**Authors:** Tomoki Nakamura, Akihiko Matsumine, Kunihiro Asanuma, Takao Matsubara, Akihiro Sudo

**Affiliations:** 1 Department of Orthopaedic Surgery, Mie University Graduate School of Medicine Tsu Japan

**Keywords:** Benign bone tumor, Proximal femur, Treatment, Reinforcement

## Abstract

*Purpose*: The proximal femur is one of the most common locations for benign bone tumors and tumor like conditions. We describe the clinical outcomes of the surgical treatment of benign lesions of the proximal femur including femoral neck using compression hip screw and synthetic bone graft.

*Methods*: Thirteen patients with benign bone tumors or tumor like conditions of the proximal femur including femoral neck were surgically treated. Their average age at the time of presentation was 35 years and the average follow-up time was 76 months.

*Results*: The average intraoperative blood loss was 1088 mL and intraoperative blood transfusion was required in eight patients. The average operative time was 167 minutes. All patients required one week and 12 weeks after surgery before full weight-bearing was allowed. All patients had regained full physical function without pain by the final follow-up. No patient sustained a pathological fracture of the femur following the procedure. All patients achieved partial or complete radiographic consolidation of the lesion within one year except one patient who developed a local tumor recurrence in 11 months. Post-operative superficial wound infection was observed in one patient, which resolved with intravenous antibiotics. Chronic hip pain was observed in one patient due to the irritation of tensor fascia lata muscle by the tube plate.

*Conclusion*: We suggest that the treatment of benign bone lesion of the proximal femur using compression hip screw and synthetic bone graft is a safe and effective method.

## Introduction

The proximal femur is one of the most common locations for benign bone tumors and tumor like conditions [1]. Surgical treatment may be required in patients with pathological fractures or aggressive benign tumors, such as giant cell tumor. Symptomatic patients complaining of recurrent pain or showing abnormal gait patterns due to bone tumors are also considered for surgical treatment. There are several treatment options for benign bone tumors of the proximal femur: curettage and bone grafting with or without internal fixation [1–5]. We describe the clinical outcomes of the surgical treatment of benign lesions of the proximal femur including the femoral neck using a compression hip screw and synthetic bone graft. The advantage of this method is that it offers immediate structural support and rapid graft incorporation and remodeling.

## Patients and methods

Sixteen patients with primary benign bone tumors or tumor like conditions of the proximal femur including the femoral neck were surgically treated at our hospital between 2002 and 2010 (Table 1). Three lesions were treated with curettage followed by implantation of calcium phosphate cement (CPC) (BIOPEX-R^®^; HOYA Technosurgical Corporation, Tokyo, Japan). Reinforcement using a compression hip screw was not performed in those three cases because one tumor included a large part of femoral head and two tumors occupied less than 50% of the diameter of the femoral neck [3, 6]. Therefore, the remaining 13 patients were analyzed. There were 10 males and three females. Their average age at the time of presentation was 35 years (21–68) and the average follow-up time was 76 months (24–157). The pathological diagnosis was fibrous dysplasia in seven patients, simple bone cyst in three, giant cell tumor in two, and chondroblastoma in one. The indications for surgical treatment were the following: tumors with impending pathological fracture, tumors causing repeated pain, and tumors with a putative expansive natural course. The 13 patients were treated with the curettage followed by implantation of CPC and/or calcium hydroxyapatite ceramic (CHA) granules (BONECERAM^®^; Olympus Corporation, Tokyo, Japan) in combination with reinforcement using a compression hip screw. These synthetic bone substitutes could be selected depending on the size of cavity. Additionally, CHA was applied to the tumors which had a risk of leaking outside of bone if CPC was injected.

**Table 1.** Patient characteristics.

Case	Age/gender	Diagnosis	Location of the tumor			Surgical position
			Head	Neck	Trochanter	
1	21/F	FD	No	Yes	Yes	Supine
2	28/M	FD	No	Yes	Yes	Supine
3	43/M	FD	No	Yes	No	Supine
4	23/M	GCT	Yes	Yes	No	Supine
5	53/M	SBC	No	Yes	No	Supine
6	41/M	FD	No	Yes	Yes	Supine
7	27/M	CB	No	Yes	Yes	Supine
8	24/F	FD	No	Yes	No	Supine
9	31/M	GCT	Yes	Yes	No	Lateral
10	31/M	SBC	No	Yes	No	Lateral
11	42/M	FD	No	Yes	Yes	Supine
12	28/M	SBC	Yes	Yes	No	Supine
13	68/F	FD	NO	Yes	Yes	Supine

F, Female; M, Male; FD, Fibrous dysplasia; GCT, Giant cell tumor, CB, Chondroblastoma; SBC, Simple bone cyst.

### Surgical procedure (Figures 1 and 2)

The operation was performed under general anesthesia with the patient either in the lateral or supine position on a radiolucent table. A lateral skin incision was made from the middle of the greater trochanter as far distally as necessary. A cortical window was made through the lateral femoral cortex under radiological control using a high-speed burr. Through a cortical window, a biopsy sample was obtained for frozen section. The definitive surgical procedure was done after pathological diagnosis of the frozen section. If the preoperative radiographic diagnosis did not correspond with the pathological diagnosis of the frozen section, a staged surgery was done. The window of the cortical bone must be large enough to allow adequate curettage of the tumor until underlying normal bone is exposed. A high-speed burr was used to remove the septum of the cavity after curettage of the bone tumor. After the lateral cortex and femoral head were reamed under radiological control, a compression hip screw was inserted. The synthetic bone was implanted in the bone defect. The weight-bearing was allowed from 1 week to 12 weeks depending on the initial strength of fixation and radiographic consolidation of the bone defect filled with CPC/CHA.

**Figure 1.**
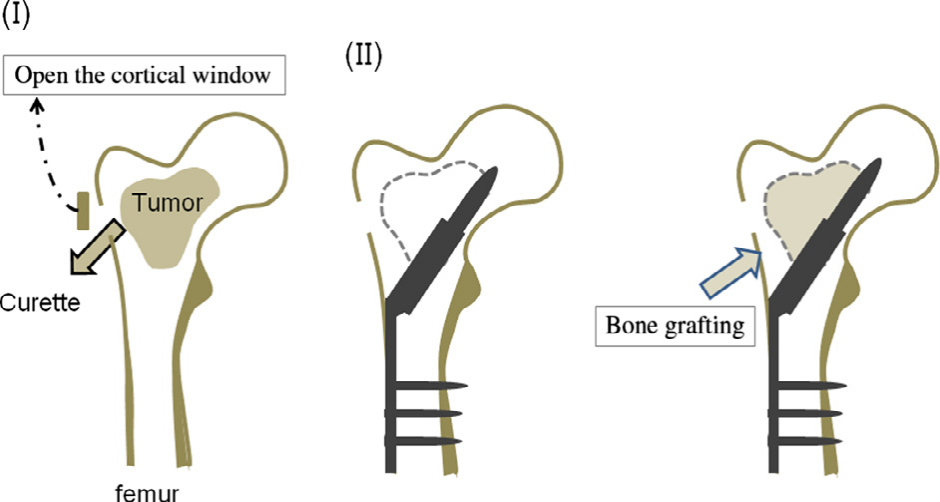
This figure shows our surgical procedure. (I) A cortical window was made at the lateral femoral cortex. Through a cortical window, a biopsy sample was obtained for frozen section. The window must be large enough to allow adequate curettage of the tumor until underlying normal bone is exposed. (II) After the lateral cortex and femoral head were reamed along with a guide wire which was inserted under radiological control, a compression hip screw was inserted. (III) The synthetic bone was implanted in the bone defect.

**Figure 2.**
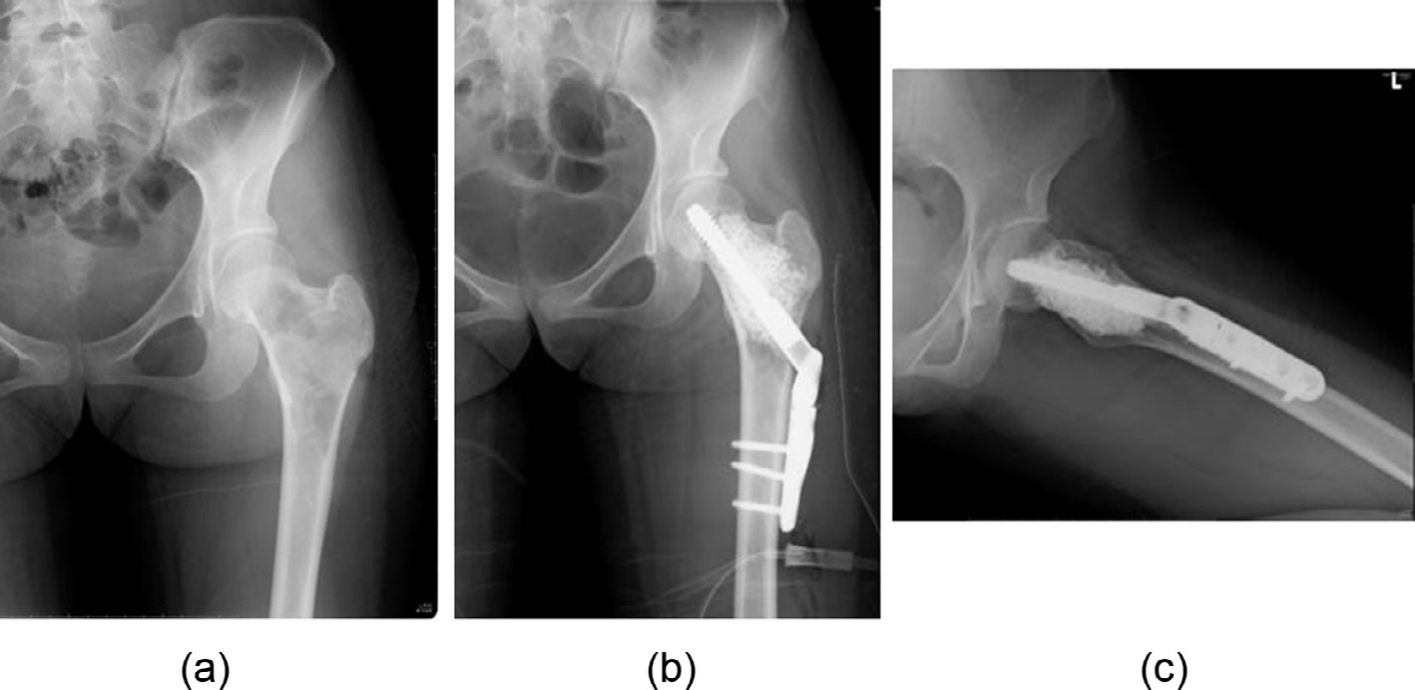
Radiograph showing fibrous dysplasia of the left proximal femur (a) (21-year-old female, Case 1). This patient with fibrous dysplasia was treated in combination with curettage of the bone tumor, the reinforcement using compression hip screw and implantation of CHA into the bone defect (b, c).

## Results (Table 2)

The average intraoperative blood loss was 1088 mL (44–3200) and intraoperative blood transfusion was required in eight patients (62%; autologous blood transfusion in five, blood component transfusion in three). The average operative time was 167 minutes (65–255). All patients were required between 1 week and 12 weeks after surgery before full weight-bearing was allowed. There was no significant difference in the duration of restriction of full weight-bearing between the patients who were treated with CPC and CHA (*p* = 0.51, Mann-Whitney U test). All patients had regained full physical function without pain by the final follow-up. None of the patients showed pain at the operation site at the time of review. No toxicity was detected in routine blood tests. No patient sustained a pathological fracture of the femur following the procedure. All achieved partial or complete radiographic consolidation of the lesion within 1 year except one patient who developed local tumor recurrence of GCT 11 months after surgery.

**Table 2.** Clinical outcome in 13 patients.

Case	Bleeding (ml)	Operative time (min)	Implantation	Reinforcement	Time to FWB	Event	Follow-up (months)
1	781	158	CHA	CHS	8 weeks	Removal of CHS	81
2	1600	255	CHA	CHS	3 weeks		60
3	580	127	CPC	CHS	1 week		42
4	3200	170	CHA	CHS	12 weeks	Local recurrence	77
5	218	135	CPC	CHS	8 weeks		129
6	1622	160	CHA/CPC	CHS	4 weeks	s/c infection	69
7	2700	158	CHA	CHS	2 weeks		73
8	444	234	CPC	CHS	2 weeks		107
9	988	165	CPC	CHS	1 week		40
10	600	200	CPC	CHS	4 weeks		24
11	1183	157	CPC	CHS	1 week		99
12	180	125	CHA/CPC	CHS	4 weeks	Removal of CHS	157
13	44	129	CHA	CHS	1 week		33

CHA, Calcium hydroxyapatite ceramic; CPC, Calcium phosphate cement; CHS, Compression hip screw; s/c, subcutaneous; FWB, Full weight-bearing.

### Complications

Post-operative superficial wound infection was observed in one patient, which resolved with intravenous antibiotics. Chronic hip pain was observed in one patient due to the irritation for tensor fascia lata muscle by the tube plate. She needed the removal of compression hip screw seven years after primary surgery. Although the side plate was easily removed, the lag screw could not be removed because of the rigid fixation between lag screw, bone, and CHA. After one and half years, chronic hip pain did not recur.

## Discussion

We have shown clinical outcomes of the treatment procedure for the proximal femoral benign bone tumor using a compression hip screw and synthetic bone graft. Some authors recommended surgical reinforcement of the proximal femur after resection of bone tumor to prevent postoperative fracture when e tumor affects more than 50% of the diameter of the femoral neck or affects at least 50% of the bone cortex of the femoral neck [3, 6]. Therefore, we used these criteria to decide the indication of compression hip screw. Shih et al. [1] reported the results of 35 patients with benign bone tumors of the femoral neck or greater trochanter area, when the bone defect was reconstructed with allogeneic cortical strut, autologous cancellous bone graft and compression hip screw via lateral approach. They showed no postoperative tumor recurrence and no complications in any patient. We used synthetic bone graft instead of allogeneic strut because allograft has a potential for transmission of infectious disease [7–9]. Furthermore, there is no available supply of allogeneic bone in Japan.

George et al. [2] reported the results of treatment for 17 benign lesions of the proximal femur with non-vascularized autologous fibular strut grafts via lateral approach. They reported that there was postoperative tumor recurrence in two patients and no postoperative pathological fracture in any patients. Although autologous bone graft has been considered to be the ideal bone substitute, its disadvantages include limited availability, the risk of tumor implantation into the donor site, and the adverse effects at the donor site such as nerve injury, bleeding, fracture and infection.

Calcium hydoxyapatite ceramic (CHA) has been used in the orthopedic field [10] during the last two decades in Japan. Immediately after surgery, there is no difference in mechanical strength between bone filled with bone substitute and bone left empty. However, because bone substitute can act as a scaffold for new bone formation, bone filled with substitute can gradually become stronger against mechanical stress than a bone defect which is left empty. Calcium phosphate cement (CPC) is an injectable biocompatible bone substitute that has been used for various applications in orthopedic surgery, thanks to attractive mixing, handling, and biological properties. CPC offered a useful bone substitute for the treatment of bone and soft tissue tumors [11]. The compressive strength of the cured materials is about 65 MPa by 3 days after mixing, reaching a final strength of >70 MPa by 1 week after mixing [12, 13]. These synthetic bone substitutes could be selected depending on the size of cavity. Therefore, we believe that full weight-bearing could be allowed within a few weeks in patients who were treated with CPC in combination with compression hip screw, even if there is large cavity at the trochanteric and femoral neck lesions after insertion of lag screw. In ten (78%) out of 13 patients with a compression hip screw, full weight-bearing was allowed within 4 weeks and there was no postoperative fracture. This duration of restriction of weight-bearing in our cases was shorter than that in previous reports (Shin et al. [1], six weeks; George et al. [2], 14 weeks). In addition, we also suggest that care should be taken when allowing early full weight-bearing in patients with tumors within the femoral head due to the risk of cut-out of the lag screw.

Some authors reported that anterior approach has an advantage of local tumor control [14]. They suggested that this approach allows complete curettage and exposure of the femoral neck and articular cartilage of the distal femoral head without dislocation. However, the lateral approach did not cause an increased rate of local recurrence in our present study. The anterior approach would have an advantage for the tumor localized in femoral head, because curettage through the lateral femoral window is difficult due to a long distance to femoral head. On the other hand, the anterior approach has an increased risk of the injury to the lateral circumflex femoral artery which leads to postoperative femoral head necrosis. Furthermore, anterior approach might cause an unexpected amputation due to contamination around femoral artery if the postoperative histological diagnosis was malignant bone tumor. Therefore, we prefer the lateral approach for the curettage of proximal femoral bone tumor.

In conclusion, we suggest that the treatment of benign bone lesion of the proximal femur using compression hip screw and synthetic bone graft is a safe and effective method.

## Conflict of interest

There was no conflict of interest in this study.
